# Clinical predictive value of the initial neutrophils to lymphocytes and platelets ratio for prognosis of sepsis patients in the intensive care unit: a retrospective study

**DOI:** 10.3389/fmed.2024.1351492

**Published:** 2024-01-18

**Authors:** Jinhui Zhang, Qun Zhao, Zhenkui Hu

**Affiliations:** Department of Critical Care Medicine, The Affiliated Hospital, Jiangsu University, Zhenjiang, China

**Keywords:** neutrophils to lymphocytes and platelets ratio, sepsis, acute kidney injury, biomarker, mortality

## Abstract

**Background:**

Neutrophils to lymphocytes and platelets (N/LP) ratio has been confirmed as an indirect marker of inflammation. In this study, we aimed to further evaluate the prognostic significance of the N/LP ratio in sepsis patients admitted to the ICU.

**Methods:**

Sepsis patients from the Affiliated Hospital of Jiangsu University were retrospectively enrolled from January 2015 and July 2023. The primary outcomes were 30/60 days mortality. The secondary outcomes included the incidence of AKI, vasoactive drug, CRRT, invasive ventilation, length of ICU stay, length of hospital stay and ICU mortality.

**Results:**

A total of 1,066 sepsis patients were enrolled with a median age of 75.0 (66.0, 85.0) years, and 62.5% of them being male. The 30 days and 60 days mortality rates were found to be 28.7 and 34.0%, respectively, while the incidence of AKI was 45.2%. Based on their N/LP ratios, we classified the sepsis patients into three groups: low, middle, and high, consisting of 266, 534, and 266 patients, respectively. According the Cox proportional hazard model, the middle and high N/LP groups were associated with a 1.990/3.106-fold increase in 30 days mortality risk and a 2.066/3.046-fold increase in 60 days mortality risk compared with the low N/LP group. Besides, multivariate logistic regression model suggested that the risk of AKI occurrence increased 2.460 fold in the high group compared to the low group. However, through subgroup analyses, we observed substantial variations in the association between N/LP ratios and 30/60 days mortality rates as well as the incidence of AKI among different populations. Notably, the N/LP ratio measured at ICU admission exhibited a higher AUC for predicting 30/60 days mortality (0.684/0.687). Additionally, we observed a good predictive power for the occurrence of AKI (AUC: 0.645) using the N/LP ratio measured at sepsis prognosis. Regarding the other secondary outcomes, the N/LP ratio was associated with disease severity in sepsis patients, including the need for vasoactive drugs, length of ICU stay, and ICU mortality.

**Conclusion:**

The N/LP ratio at ICU admission was found to have a significant independent association with 30/60 days mortality and the incidence of AKI in sepsis patients.

## Introduction

Sepsis, a life-threatening condition characterized by organ dysfunction caused by an uncontrolled immune response to infection, remains a significant threat to human survival (1). According to previous epidemiological studies, the global incidence of sepsis is approximately 50 million cases per year, with approximately 11 million deaths annually (2). In China, it is estimated that 20.6% of patients in the intensive care unit (ICU) suffer from sepsis, and over 35.5% of them succumb to the condition (3). Despite extensive research efforts focused on understanding the pathogenesis, early diagnosis, and clinical treatment of sepsis, the mortality rate among ICU patients with sepsis remains alarmingly high (4, 5). Therefore, identifying applicable prognostic factors for sepsis patients is crucial to facilitate timely interventions and improve patient outcomes.

Previous studies had identified several biomarkers such as oncostatin M (OSM), apoptosis inhibitor of the macrophage (AIM/CD5L), interleukin-26 (IL-26), interleukin-17D (IL-17D), interleukin-37 (IL-37), and growth differentiation factor-15 (GDF-15) as potential predictors of sepsis prognosis (6–11). However, most of these markers were costly and not feasible for routine clinical practice. In light of this, it is worth noting that systemic inflammatory responses play a significant role in the development and progression of sepsis (12). The neutrophils to lymphocytes and platelets (N/LP) ratio, a readily available inflammation marker, had emerged as a low-cost alternative that can be assessed through routine blood counts (13). The N/LP ratio had shown utility in reflecting patients’ inflammatory status and had been identified as a risk factor for adverse outcomes in various conditions, including Corona Virus Disease 2019 (COVID-19) and the occurrence of acute kidney injury (AKI) following abdominal and cardiovascular surgeries (14–16). However, the relationship between N/LP ratios and adverse outcomes in sepsis patients has received limited attention.

Sepsis is a severe infectious disease characterized by dysregulation of the body’s immune system and activation of inflammatory response. During the onset of sepsis, immune cells such as neutrophils and lymphocytes participate in the immune process, and platelets also play an important role in the inflammatory response (17). Studies had shown that the neutrophil-to-lymphocyte ratio (NLR) was considered an indicator of the equilibrium between innate and adaptive immune responses. It had been extensively investigated as a prognostic factor in numerous inflammatory diseases, including sepsis (18–20). However, in cases of sepsis, the activation of inflammation and coagulation systems can lead to platelet aggregation and decrease (21). Consequently, monitoring the N/LP ratio may provide some reference for evaluating the prognosis of sepsis. Currently, there is a paucity of research examining the association between N/LP ratios and the prognosis of sepsis patients in the ICU. Therefore, the main objective of this study was to investigate the prognostic value of the N/LP ratio in ICU patients with sepsis.

## Methods

### Patient selection

This retrospective analysis was conducted at the Affiliated Hospital of Jiangsu University, focusing on sepsis patients admitted between January 2015 and July 2023, including both internistic and surgical patients. The inclusion criteria comprised patients who met the Sepsis 3.0 diagnostic criteria (confirmed or suspected infection with a total SOFA score of 2 points) (1) at the time of ICU admission and were aged 18 or above. Additionally, patients needed to have complete clinical data available. Exclusion criteria included patients who were discharged or died within 24 h of ICU admission, patients with chronic kidney disease (CKD), and patients with hepatic cirrhosis. A diagnosis of CKD is confirmed if a patient presents a glomerular filtration rate (GFR) below 60 mL/min/1.73 m^2^ for three consecutive months, accompanied by alterations in any kidney injury marker or evidence from imaging tests (22). The study protocol was approved by the Ethics Committee of the Affiliated Hospital of Jiangsu University (Approval No. KY2023K1007). All patients provided written informed consent.

### Data collection

Clinical data for the included patients were extracted from electronic medical records. The collected clinical data encompassed various aspects, including demographic information [age, gender, smoking, body mass index (BMI)], associated comorbidities [hypertension, diabetes, coronary artery disease, chronic obstructive pulmonary disease (COPD), cerebral infarction], infection pathogens (gram-positive bacteria, gram-negative bacteria, fungus, virus), infection locations (multisite infection, lower respiratory infection, gastrointestinal infection, intra-abdominal infection, genitourinary tract infection, bacteremia, skin and soft tissue infection), laboratory test variables [white blood cell (WBC), neutrophil (Neu), lymphocyte (Lym), platelet (PLT), hemoglobin (Hb), C-reactive protein (CRP), albumin (Alb), creatinine, glucose, blood urea nitrogen (BUN), uric acid, endotoxin, procalcitonin (PCT), prothrombin time (PT), D-dimer, potassium, sodium, lactate], severity scores [Acute Physiology and Chronic Health Evaluation II (APACHE II) score, Sequential Organ Failure Assessment (SOFA) score], therapies administered [use of vasoactive drug, continuous renal replacement therapy (CRRT), invasive ventilation] and endpoints (incidence of AKI, length of ICU stay, length of hospital stay, 30 days mortality, 60 days mortality, and ICU mortality). The worst values of the above laboratory markers were collected within 24 h of ICU admission. AKI was diagnosed according to the criteria set by the Kidney Disease Improving Global Outcomes (KDIGO) guidelines (23). Specifically, AKI was defined as an increase in creatinine levels to at least 1.5 times the baseline within the prior 7 days or an absolute increase of at least 0.3 mg/dL within 48 h. It is important to note that the diagnosis of AKI was made after the patient’s admission to the ICU.

The N/LP ratio was calculated using the equation: Neutrophil count × 100/(lymphocyte count × platelet count) (14). Patients were then categorized into three groups based on their N/LP ratio quartile range: low (N/LP <= 5.68, <=25th percentile), middle (5.68 <= N/LP <= 10.4128, 25th–75th percentile), and high (N/LP >= 28.41, >=75th percentile).

### Outcomes

The primary outcomes of interest in this study were the 30 days mortality and 60 days mortality. The secondary outcomes included the incidence of AKI, use of vasoactive drugs, CRRT, invasive ventilation, length of ICU stay, length of hospital stay, and ICU mortality.

### Statistical analysis

For continuous variables, descriptive statistics such as mean with standard deviation or median with interquartile ranges (IQR) were calculated. The one-way ANOVA test or nonparametric Kruskal–Wallis test was used to compare continuous variables between groups. Categorical variables were presented as frequencies and percentages, and comparisons between groups were made using chi-square or Fisher exact tests. To evaluate the mortality of sepsis patients based on the N/LP ratio, Kaplan–Meier curves, Cox proportional hazards models, and cubic spline regression analyses were performed. Kaplan–Meier curves were used to analyze cumulative survival probabilities at 30 days and 60 days mortality. Multivariate Cox proportional hazard models were employed to examine the relationship between the N/LP ratio and 30 days and 60 days mortality, adjusting for various covariates in different models. Hazard ratios (HR) and 95% confidence intervals (CI) were reported. Model 1 did not include any covariate adjustments, while Model 2 adjusted for age, hypertension, diabetes, Alb, creatinine, BUN, uric acid, SOFA score, CRRT, and invasive ventilation. Model 3 adjusted for age, BMI, smoking, hypertension, diabetes, Hb, CRP, Alb, creatinine, BUN, uric acid, PT, D-dimer, potassium, APACHE II score, SOFA score, vasoactive drug, CRRT, invasive ventilation, and incidence of AKI. Additionally, cubic spline regression analysis was used to visualize the association between the N/LP ratio and mortality risk, with five equally distributed knots along the N/LP ratio curve, placed at 5, 27.5, 50, 72.5, and 95 percentiles for the N/LP ratio. The reference point was set at the 5.68 (25th). The association between the N/LP ratio and the incidence of AKI was investigated through logistic regression models with different degrees of covariate adjustment. Model 1 was an univariate analysis without adjusting for any covariates. Model 2 adjusted for age, gender, hypertension, diabetes, intra-abdominal infection, genitourinary tract infection, WBC, potassium, and lactate level. Model 3 adjusted for age, gender, BMI, hypertension, diabetes, coronary artery disease, gram-negative bacteria, intra-abdominal infection, genitourinary tract infection, bacteremia, WBC, potassium, sodium, and lactate level. Cubic spline regression analysis was also employed to explore the relationship between the N/LP ratio and the occurrence of AKI. Additionally, we performed a stratified analysis to examine whether the N/LP ratio had any association with 30/60 days mortality in specific subgroups based on age, gender, hypertension, diabetes, SOFA score, and AKI. We also investigated whether the N/LP ratio was associated with the occurrence of AKI in subgroups stratified by age, gender, hypertension, diabetes, SOFA score, and lactate level. Furthermore, we estimated the predictive value of the N/LP ratio for 30/60 days mortality and the incidence of AKI using the ROC analysis. To assess the association between the N/LP ratios and in-hospital adverse outcomes, multivariate binary logistic or Cox or linear regression analysis was employed. All statistical analyses were conducted using IBM SPSS 26.0, GraphPad Prism 9.0, and R version 4.2.0. Statistical significance was defined as a *p*-value less than 0.05 (two-sided).

## Results

### Patient characteristics

**Figure 1:**
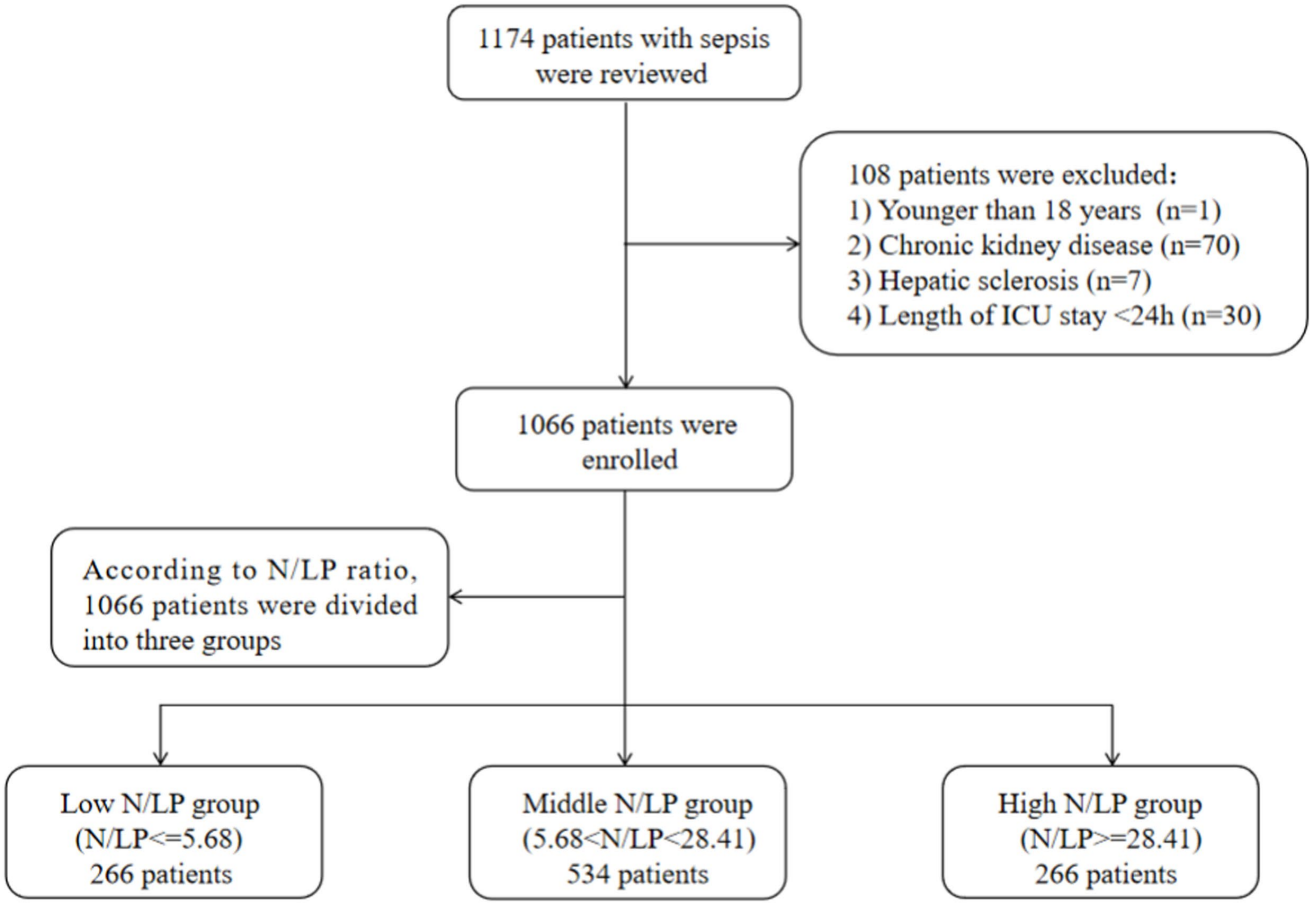
Flow diagram of the study. ICU, intensive care unit.

**Figure 2:**
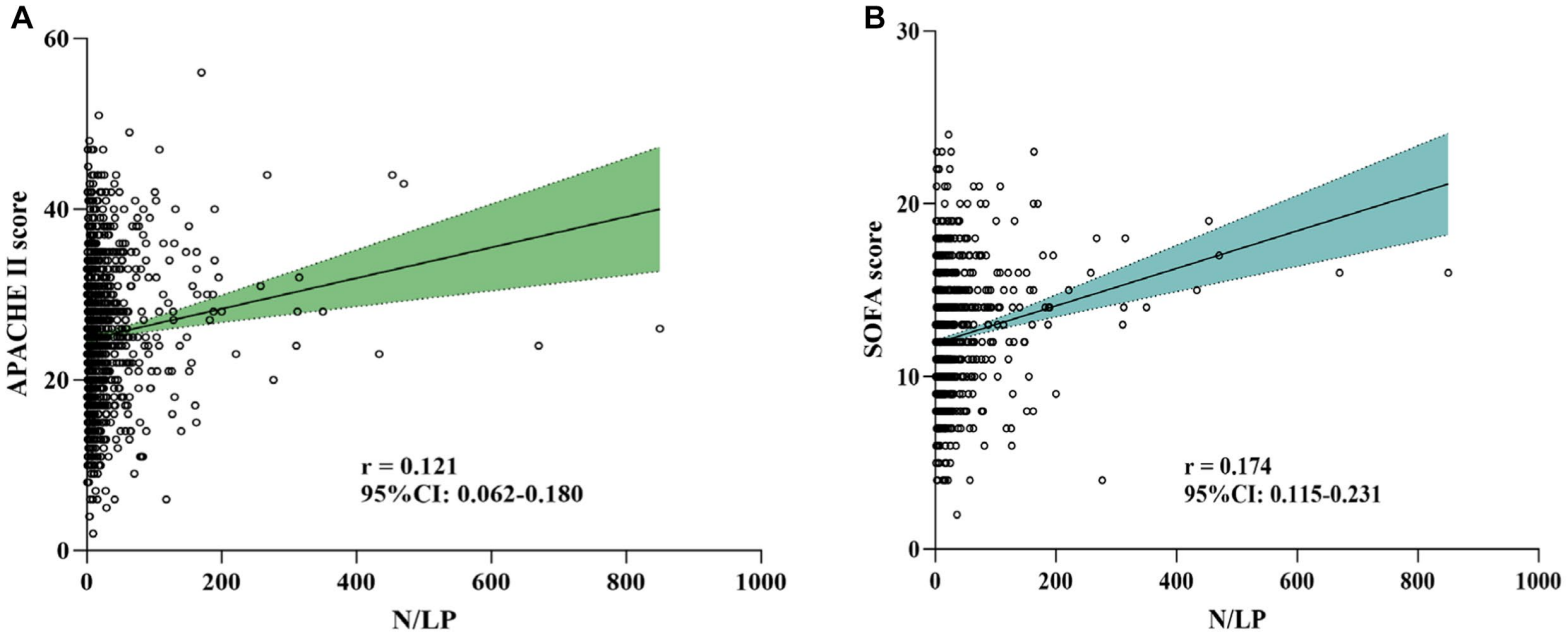
Association between N/LP ratios and APACHE II score **(A)** and SOFA score **(B)** using Spearman’s analysis. APACHE II, acute physiology and chronic health evaluation II; SOFA, sequential organ failure assessment.

In this study, a total of 1,066 patients were included (Figure 1). The distribution of the N/LP ratio was summarized as follows: the 25th percentile, median, and 75th percentile were 5.68, 12.14, and 28.41, respectively. Based on predefined grouping criteria, 266 patients were categorized into the low N/LP group, 534 into the middle N/LP group, and 266 into the high N/LP group. Table 1 presented the baseline characteristics of the patients. The median age was 75.0 years (interquartile range: 66.0, 85.0), with 666 (62.5%) being male. Among the enrolled patients, 209 (19.6%) were smokers, and the median BMI was 22.60 kg/m^2^ (interquartile range: 19.92, 25.38). Hypertension (51.7%) was the most prevalent comorbidity, followed by diabetes (36.9%), cerebral infarction (14.4%), coronary artery disease (10.3%), and COPD (7.7%). Regarding the source of infection, the high N/LP group had a higher proportion of gram-negative bacteria (*p* = 0.005) and multisite infection (*p* < 0.001) compared to the other groups. Several laboratory parameters were significantly different among the N/LP groups. The high N/LP group had significantly higher levels of WBC, Neu, CRP, creatinine, BUN, uric acid, PCT, PT, D-dimer, and lactate (*p* < 0.001). Conversely, Lym, Hb, PLT, and Alb were significantly lower in the high N/LP group (*p* < 0.001). As anticipated, the high N/LP group demonstrated significantly higher APACHE II score and SOFA score when compared to the other groups (*p* < 0.001, *p* < 0.001, respectively). Spearman’s analysis was conducted to examine the correlation between N/LP ratios and APACHE II score and SOFA score (Figure 2). The resulting correlation coefficients were as follows: N/LP and APACHE II score = 0.121 (*p* < 0.001), N/LP and SOFA score = 0.174 (*p* < 0.001).

**Table 1:** Baseline demographic and clinical characteristics by different N/LP ratios in sepsis patients.

Variables	Overall	Low N/LP group (N/LP < =5.68)	Middle N/LP group (5.68 < N/LP < 28.41)	High N/LP group (N/LP > =28.41)	*p*
*N*	1,066	266	534	266	
Age, years	75.0 (66.0, 85.0)	75.0 (62.0, 85.0)	75.5 (66.0, 85.0)	75.0 (66.0, 84.0)	0.681
Male, *n* (%)	666 (62.5)	169 (63.5)	336 (62.9)	161 (60.5)	0.740
BMI, kg/m^2^	22.49 (20.05, 25.15)	23.41 (20.02, 26.03)	22.49 (20.00, 24.97)	22.04 (20.20, 24.33)	0.049
Smoking, *n* (%)	209 (19.6)	52 (19.5)	103 (19.3)	54 (20.3)	0.947
**Comorbidities, *n* (%)**					
Hypertension	551 (51.7)	148 (55.6)	275 (51.5)	128 (48.1)	0.220
Diabetes	287 (26.9)	78 (29.3)	138 (25.8)	71 (26.7)	0.576
Coronary artery disease	110 (10.3)	59 (22.2)	55 (10.3)	26 (9.8)	0.906
COPD	82 (7.7)	21 (7.9)	41 (7.7)	20 (7.5)	0.987
Cerebral infarction	153 (14.4)	37 (13.9)	93 (17.4)	23 (8.6)	0.004
**Infection pathogens, *n* (%)**					
Gram-positive bacteria	132 (12.4)	33 (12.4)	68 (12.7)	31 (11.7)	0.909
Gram-negative bacteria	317 (29.7)	72 (27.1)	145 (27.2)	100 (37.6)	0.005
Fungus	76 (7.1)	11 (4.1)	40 (7.5)	25 (9.4)	0.056
Virus	58 (5.4)	9 (3.4)	35 (6.6)	14 (5.3)	0.175
**Infection sites, *n* (%)**					
Multisite Infection	115 (10.8)	13 (4.9)	55 (10.3)	47 (17.7)	<0.001
Lower respiratory infection	421 (39.5)	101 (38.0)	225 (42.1)	95 (35.7)	0.182
Gastrointestinal infection	8 (0.8)	2 (0.8)	3 (0.6)	3 (1.1)	0.683
Intra-abdominal infection	362 (34.0)	111 (41.7)	172 (32.2)	79 (29.7)	0.007
Genitourinary tract infection	66 (6.2)	11 (4.1)	34 (6.4)	21 (7.9)	0.193
Bacteremia	11 (1.0)	0 (0)	5 (0.9)	6 (2.3)	0.014
Skin and soft tissue infection	83 (7.8)	28 (10.5)	40 (7.5)	15 (5.6)	0.103
**Laboratory tests**					
WBC *10^9^/L	11.40 (7.40, 17.13)	8.65 (5.28, 12.30)	11.90 (7.90, 17.20)	14.20 (9.90, 20.43)	<0.001
Neu *10^9^/L	10.10 (6.38, 15.60)	6.60 (3.90, 10.33)	10.55 (7.10, 15.75)	13.60 (9.18, 19.73)	<0.001
Lym *10^9^/L	0.6 (0.3, 0.9)	1.1 (0.7, 1.5)	0.5 (0.4, 0.8)	0.3 (0.2, 0.5)	<0.001
Hb, g/dL	115 (97, 129)	121 (103, 135)	113 (98, 129)	111 (90, 125)	<0.001
PLT *10^9^/L	150 (95, 214)	214 (169, 284)	154 (110, 211)	81 (48, 118)	<0.001
CRP, mg/L	103.15 (42.28, 161.45)	63.05 (13.78, 117.20)	107.40 (46.18, 162.10)	132.30 (73.73, 190.30)	<0.001
Alb, g/L	28.20 (24.10, 33.20)	30.30 (25.18, 34.83)	28.10 (24.50, 33.10)	26.80 (22.70, 31.15)	<0.001
Creatinine, μmol/L	91.75 (63.20, 150.28)	77.55 (55.20, 120.85)	85.60 (61.40, 141.90)	129.60 (78.88, 215.93)	<0.001
Glucose, mmol/L	8.20 (6.54, 11.69)	7.92 (6.45, 10.77)	8.23 (6.57, 11.95)	8.52 (6.54, 11.97)	0.210
BUN, mmol/L	8.81 (5.98, 13.96)	7.00 (4.92, 10.43)	8.50 (5.96, 12.68)	12.81 (8.33, 19.07)	<0.001
Uric acid, μmol/L	287.80 (190.05, 411.73)	269.55 (184.48, 374.93)	276.05 (183.65, 393.50)	343.35 (230.43, 488.93)	<0.001
Endotoxin, EU/mL	0.10 (0.04, 0.34)	0.07 (0.03, 0.26)	0.11 (0.05, 0.36)	0.08 (0.03, 0.33)	0.129
PCT, ng/mL	3.95 (0.39, 26.00)	0.97 (0.16, 10.25)	2.70 (0.39, 22.75)	19.00 (2.78, 47.25)	<0.001
PT, s	13.5 (12.4, 15.0)	13.0 (12.0, 14.2)	13.5 (12.3, 14.8)	14.6 (13.1, 16.73)	<0.001
D-dimer, mg/L	4.27 (2.13, 8.45)	3.10 (1.63, 6.00)	4.32 (2.11, 8.20)	6.09 (3.06, 12.61)	<0.001
Potassium, mmol/L	3.68 (3.29, 4.16)	3.72 (3.28, 4.15)	3.67 (3.32, 4.11)	3.71 (3.29, 4.28)	0.618
Sodium, mmol/L	135.20 (131.40, 139.00)	135.55 (131.80, 139.00)	135.30 (131.60, 139.00)	134.55 (130.13, 138.80)	0.141
Lactate, mmol/L	2.1 (1.4, 3.8)	1.8 (1.2, 2.8)	2.0 (1.4, 3.6)	2.8 (1.8, 5.1)	<0.001
**Severity scoring**					
APACHE II score	25 (20, 30)	23 (18, 30)	25 (19, 30)	28 (22, 33)	<0.001
SOFA score	12 (10, 14)	11 (9, 13)	12 (10, 14)	14 (11, 15)	<0.001

BMI, body mass index; COPD, chronic obstructive pulmonary disease; WBC, white blood cell; Neu, neutrophil; Lym, lymphocyte; PLT, platelet; CRP, C-reactive protein; Alb, albumin; BUN, blood urea nitroge; PCT, procalcitonin; PT, prothrombin time; APACHE II, acute physiology and chronic health evaluation II; SOFA, sequential organ failure assessment.

### Patient outcomes

Among the 1,066 patients included in the study, 715 (67.1%) received vasoactive drugs and 720 (67.5%) required invasive ventilation. AKI was diagnosed in 483 (45.2%) patients, with 74 (6.9%) requiring CRRT. During the hospital admission, a total of 306 (28.7%) deaths occurred within 30 days, and 362 (34.0%) deaths occurred within 60 days (Table 2). Additionally, the median length of ICU stay was 6 days (interquartile range: 3, 12), and the median length of hospital stay was 17 days (interquartile range: 11, 25). Among the patients admitted to the ICU, 346 (32.5%) individuals died during their ICU admission.

**Table 2:** Patient outcomes based on the N/LP ratio.

Variables	Overall	Low N/LP group (N/LP < =5.68)	Middle N/LP group (5.68 < N/LP < 28.41)	High N/LP group (N/LP > =28.41)	*p*
**Primary outcomes**					
30 days mortality, *n* (%)	306 (28.7)	31 (11.7)	151 (28.3)	124 (46.6)	<0.001
60 days mortality, *n* (%)	362 (34.0)	36 (13.5)	185 (34.6)	141 (53.0)	<0.001
**Secondary outcomes**					
AKI, *n* (%)	483 (45.3)	83 (31.2)	228 (42.7)	172 (64.7)	<0.001
CRRT, *n* (%)	74 (6.9)	7 (2.6)	31 (5.8)	36 (13.5)	<0.001
Vasoactive drug, *n* (%)	715 (67.1)	139 (52.3)	353 (66.1)	223 (83.8)	<0.001
Invasive ventilation, *n* (%)	720 (67.5)	169 (63.5)	374 (70.0)	177 (66.5)	0.166
Length of ICU stay, days	6 (3, 12)	5 (2, 10)	6 (3, 12)	7(4, 13)	<0.001
Length of hospital stay, days	17 (11, 25)	17 (12, 25)	17 (11, 26)	15 (9, 24)	0.047
ICU mortality, *n* (%)	346 (32.5)	37 (13.9)	172 (32.2)	137 (51.5)	<0.001

AKI, acute kidney injury; CRRT, continuous renal replacement therapy; ICU, intensive care unit.

### The relationship between N/LP ratios and 30/60 days mortality

**Figure 3:**
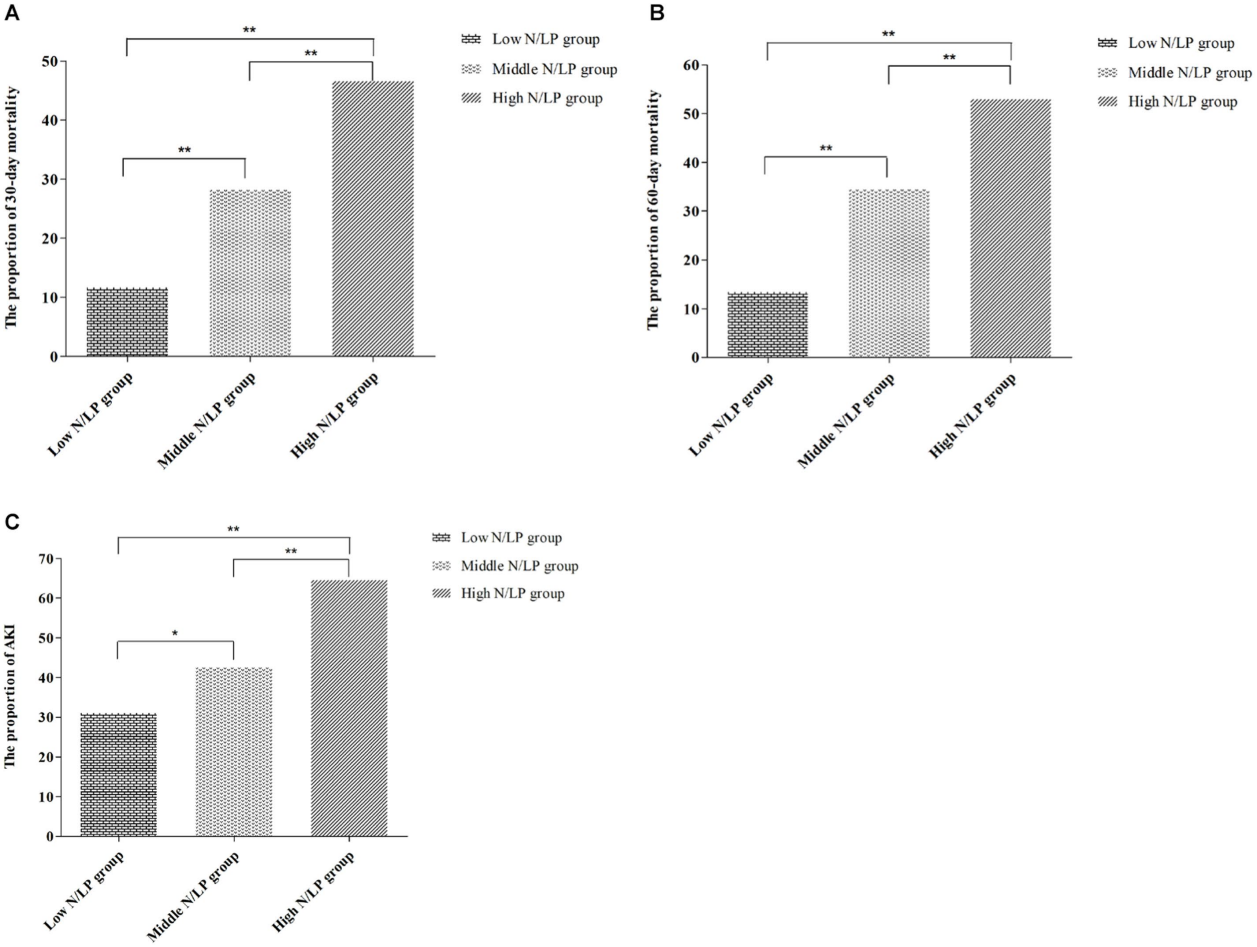
The 30/60 days mortality and the proportion of AKI in different N/LP groups. **(A)** The 30 days mortality of sepsis patients in each N/LP group were 11.7% (low N/LP group), 28.3% (middle N/LP group), and 46.6% (high N/LP group); **(B)** The 60 days mortality of sepsis patients in each N/LP group were 13.5% (low N/LP group), 34.6% (middle N/LP group), and 53.0% (high N/LP group); **(C)** The proportion of AKI in each N/LP group were 31.2% (low N/LP group), 42.7% (middle N/LP group), and 64.7% (high N/LP group). Statistical analysis by chi-square test; *p < 0.05 and **p < 0.001. AKI, acute kidney injury.

**Figure 4:**
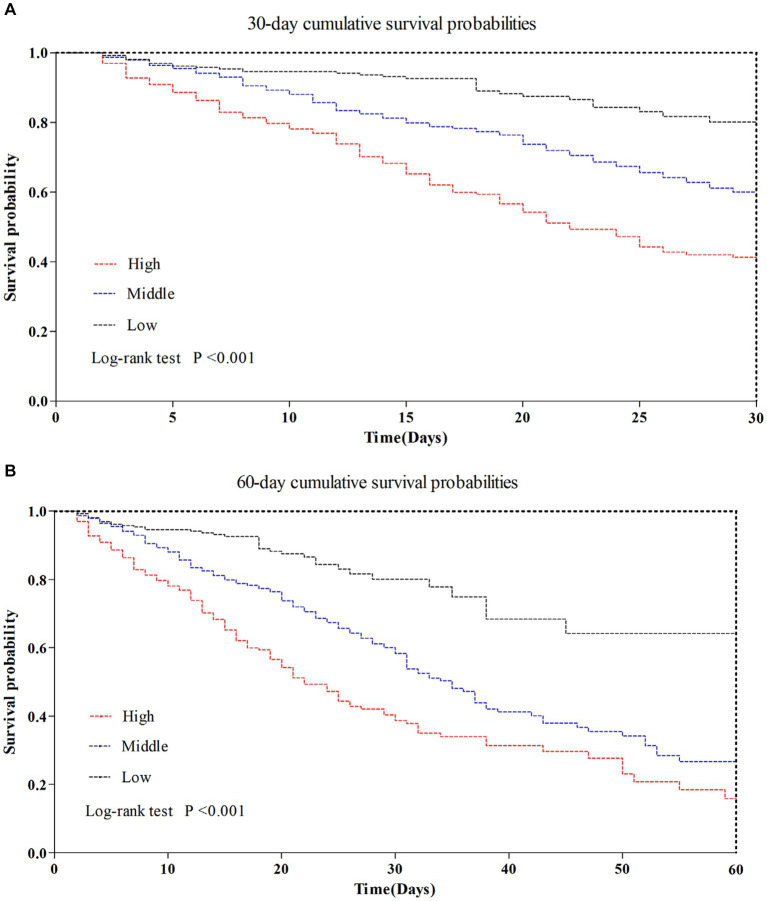
Kaplan–Meier analysis for 30/60 days survival basing on the N/LP ratio. **(A)** Kaplan–Meier analysis for 30 days survival basing on the N/LP ratio at ICU admission. **(B)** Kaplan–Meier analysis for 60 days survival basing on the N/LP ratio at ICU admission.

**Figure 5:**
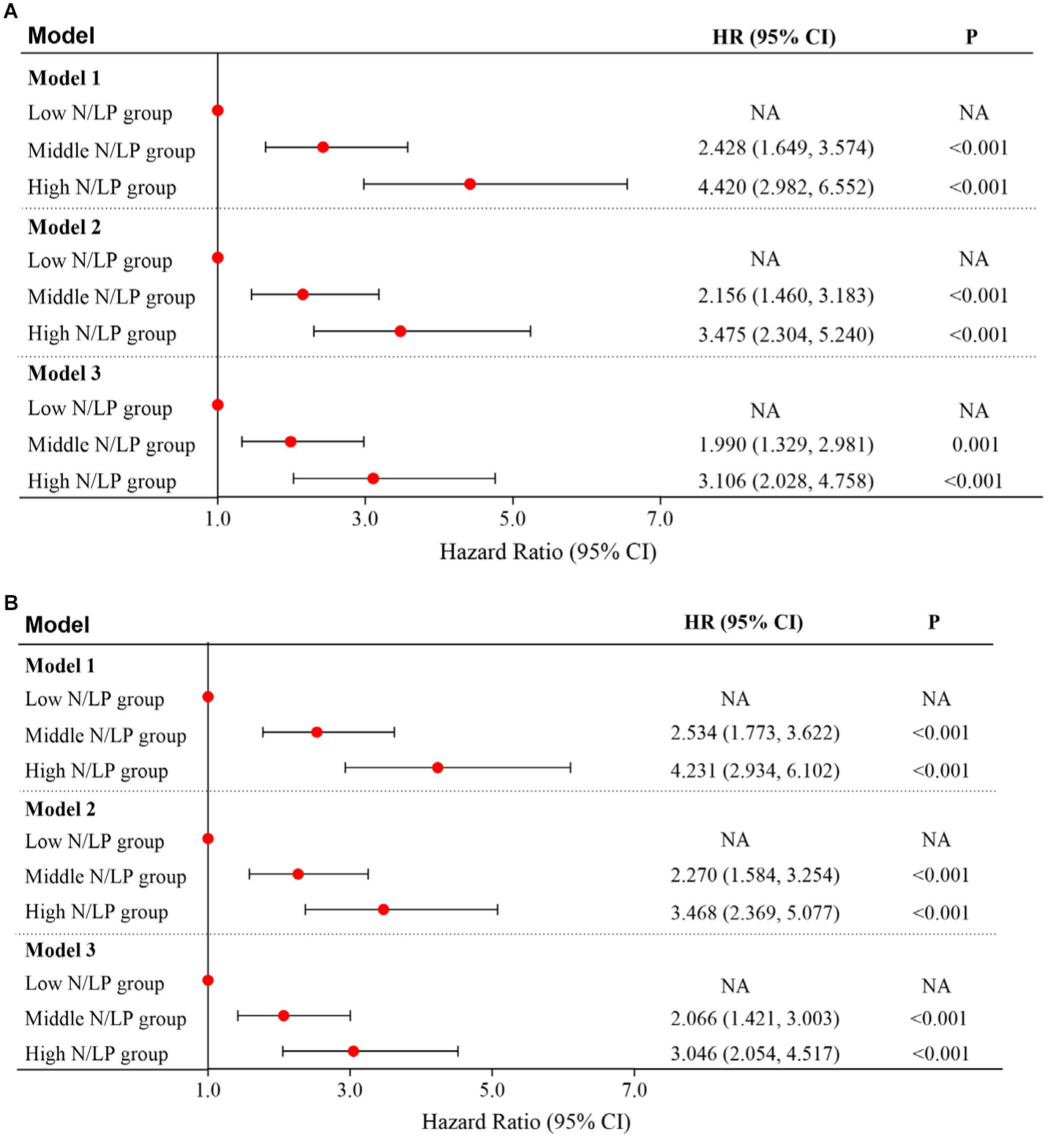
Multivariate cox proportional hazard model for 30 days **(A)** and 60 days **(B)** mortality based on N/LP ratio; Model 1: univariate analysis; Model 2: adjusted for age, hypertension, diabetes, Alb, creatinine, BUN, uric acid, SOFA score, CRRT, and invasive ventilation; Model 3: adjusted for age, BMI, smoking, hypertension, diabetes, Hb, CRP, Alb, creatinine, BUN, uric acid, PT, D-dimer, potassium, APACHEII score, SOFA score, vasoactive drug, CRRT, invasive ventilation, and incidence of AKI. BMI, body mass index; Hb, hemoglobin; CRP, C-reactive protein; Alb, albumin; BUN, blood urea nitroge; PT, prothrombin time; APACHE II, acute physiology and chronic health evaluation II; SOFA, sequential organ failure assessment; CRRT, continuous renal replacement therapy; ICU, intensive care unit; AKI, acute kidney injury.

**Figure 6:**
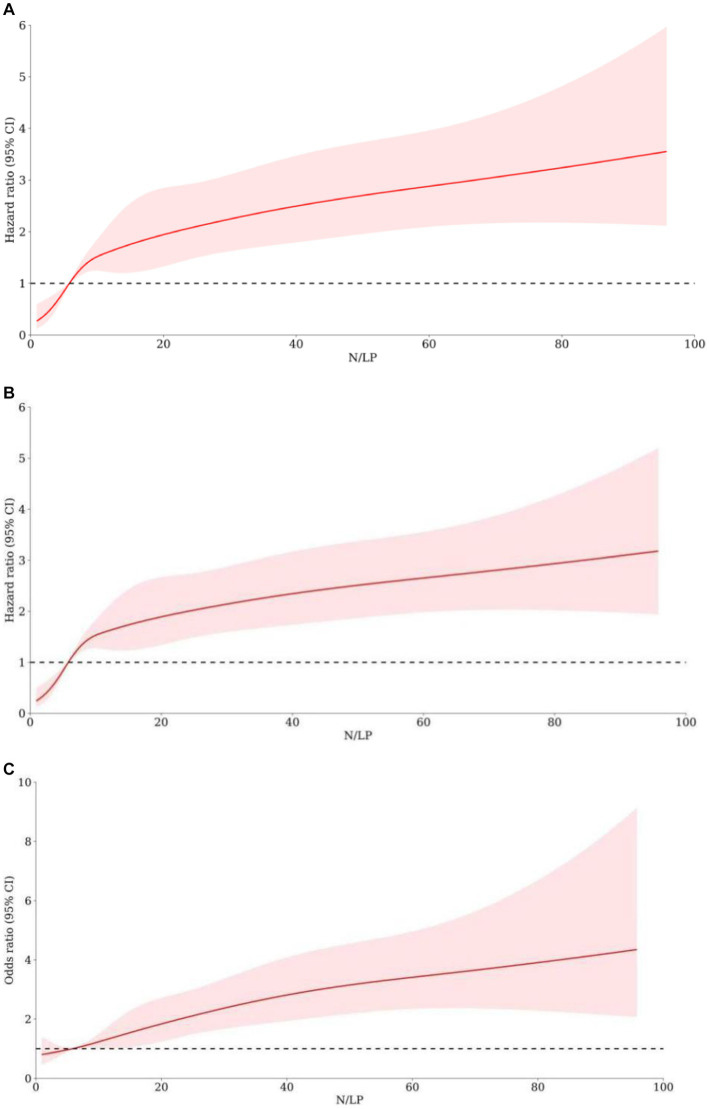
The association between N/LP ratios and the adverse outcomes risk by using cubic spline regression analyses. The 30 days mortality risk **(A)**, 60 days mortality risk **(B)**, and AKI occurrence **(C)** were analyzed by using cubic spline regression basing on the N/LP ratio. AKI, acute kidney injury.

As indicated in Table 2, there was a significant variation in 30/60 days mortality within each N/LP group. The mortality rates increased progressively with higher N/LP ratios (Figures 3A,B). Furthermore, the cumulative incidence curves generated using the Kaplan–Meier method and the log-rank test (*p* < 0.001) displayed the same pattern (Figure 4). To further investigate the impact of different N/LP ratios on 30/60 days mortality in sepsis patients, univariate and multivariate Cox proportional hazards regression analyses were conducted (Figures 5A,B). Based on the results of the univariate Cox analysis, variables such as age, BMI, smoking, hypertension, diabetes, Hb, CRP, Alb, creatinine, BUN, uric acid, PT, D-dimer, potassium, APACHE II score, SOFA score, vasoactive drug, CRRT, invasive ventilation, and incidence of AKI were included in the multivariate analysis. The findings demonstrated that a high N/LP ratio was associated with an increased HR for both 30 days and 60 days mortality in sepsis patients. This association remained consistent in all three models. In Model 3, after adjusting for all twenty-one covariates, the middle and high N/LP ratio groups had a 1.990/3.106-fold higher risk of 30 days mortality (95% CI: 1.329, 2.981; 95% CI: 2.028, 4.758, respectively) and a 2.066/3.046-fold higher risk of 60 days mortality (95% CI: 1.421, 3.003; 95% CI: 2.054, 4.517, respectively) compared to the low N/LP group. Moreover, the relationship between N/LP ratios and 30/60 days mortality risk was assessed using cubic spline regression analyses (Figures 6A,B). The HR for 30/60 days mortality significantly increased when the N/LP ratio upon ICU admission exceeded or equaled 5.68. Furthermore, the risk of 30/60 days mortality substantially escalated with higher N/LP ratios.

### The incidence of AKI in various N/LP groups

**Figure 7:**
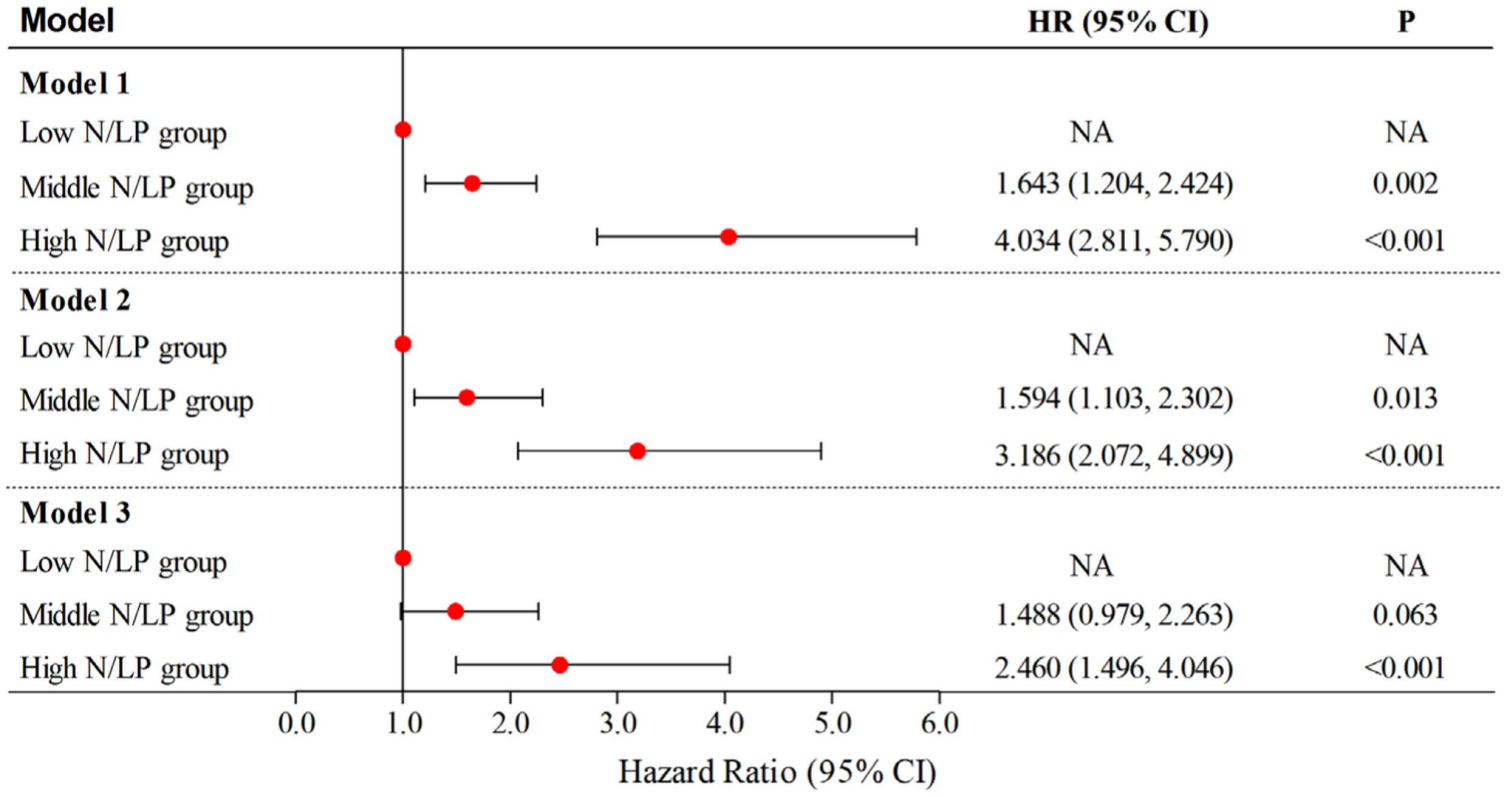
Multivariate logistic regression analyses for AKI occurrence based on N/LP ratio; Model 1: univariate analysis; Model 2: adjusted for age, gender, hypertension, diabetes, intra-abdominal infection, genitourinary tract infection, WBC, potassium and lactate level; Model 3: adjusted for age, gender, BMI, hypertension, diabetes, coronary artery disease, gram-negative bacteria, intra-abdominal infection, genitourinary tract infection, bacteremia, WBC, potassium, sodium and lactate level. BMI, body mass index; WBC, white blood cell count; AKI, acute kidney injury.

According to the data presented in Table 2, the high N/LP group showed a significantly higher proportion of patients with AKI compared to the other groups (*p* < 0.001). Furthermore, as shown in Figure 3C, the proportion of AKI cases increased with higher N/LP ratios. The results of the multivariate logistic regression analysis, summarized in Figure 7, indicated that only the high N/LP group had a significantly increased risk of AKI compared to the low NLR group (HR 2.460; 95%CI: 1.496, 4.046), despite the fact that HR increased. Additionally, the cubic spline regression analysis demonstrated a clear association between an increased N/LP ratio and a higher risk of AKI occurrence (Figure 6C).

### Subgroup analysis

We conducted subgroup analyses to investigate the correlation between different N/LP ratios and the risk of 30 days or 60 days mortality in patients with sepsis in the ICU. The results are presented in Tables 3, 4. Interaction tests were conducted to examine the potential influence of age, gender, hypertension, diabetes, SOFA score, and AKI on the risk of mortality. However, these tests yielded non-significant results for both 30 days mortality (*p* = 0.154, 0.104, 0.055, 0.967, 0.907, and 0.990) and 60 days mortality (*p* = 0.279, 0.360, 0.117, 0.897, 0.508, and 0.761). Moreover, a subgroup analysis was performed to evaluate the association between N/LP ratios and the risk of AKI in septic patients. The results are presented in Supplementary Table S1. Similarly, interaction tests were conducted to assess the potential impact of age, gender, hypertension, diabetes, SOFA score, and lactate level on the risk of AKI. However, these tests also yielded non-significant results (*p* = 0.982, 0.110, 0.237, 0.779, 0.208, and 0.415). Nevertheless, we observed that higher N/LP ratios had a noticeable impact on male patients, older individuals, and those with poor health conditions.

**Table 3:** Subgroup analysis regarding the influence of different N/LP ratios in the 30 days mortality.

Subgroups	No. 30 days mortality /No. patients	Low N/LP group (N/LP < =5.68)	Middle N/LP group (5.68 < N/LP < 28.41)	P1	High N/LP group (N/LP > =28.41)	P2	*p* for interaction
Age							0.154
<=75	117/539	Ref	2.770 (1.394, 5.505)	0.003	7.636 (3.770, 15.469)	<0.001	
>75	189/527	Ref	3.212 (1.873, 5.498)	<0.001	6.254 (3.475, 11.254)	<0.001	
Gender							0.104
Female	109/400	Ref	2.223 (1.164, 4.246)	0.014	3.798 (1.907, 7.563)	<0.001	
Male	197/666	Ref	3.629 (2.085, 6.315)	<0.001	9.514 (5.280, 17.144)	<0.001	
Hypertension							0.055
Yes	168/551	Ref	2.431 (1.456, 4.061)	0.001	4.795 (2.728, 8.430)	<0.001	
No	138/515	Ref	4.702 (2.177, 10.157)	<0.001	11.892 (5.387, 26.251)	<0.001	
Diabetes							0.967
Yes	82/287	Ref	2.679 (1.252, 5.729)	0.009	5.905 (2.624, 13.291)	<0.001	
No	224/779	Ref	3.136 (1.895, 5.190)	<0.001	6.958 (4.080, 11.868)	<0.001	
SOFA score							0.907
<=12	110/608	Ref	6.500 (2.177, 19.423)	<0.001	5.205 (1.980, 13.684)	<0.001	
>12	196/458	Ref	5.960 (2.290, 15.515)	<0.001	3.440 (1.344, 8.803)	0.007	
AKI							0.990
Yes	188/483	Ref	4.761 (2.335, 9.705)	<0.001	7.648 (3.703, 15.796)	<0.001	
No	118/583	Ref	1.921 (1.126, 3.277)	0.015	4.788 (2.586, 8.866)	<0.001	

P1: Middle N/LP group vs Low N/LP group; P2: High N/LP group vs Low N/LP group; SOFA, sequential organ failure assessment; AKI, acute kidney injury.

**Table 4:** Subgroup analysis regarding the influence of different N/LP ratios in the 60 days mortality.

Subgroups	No. 60 days mortality /No. patients	Low N/LP group (N/LP < =5.68)	Middle N/LP group (5.68 < N/LP < 28.41)	P1	High N/LP group (N/LP > =28.41)	P2	*p* for interaction
Age							0.279
<=75	139/539	Ref	3.559 (1.852, 6.840)	<0.001	8.087 (4.084, 16.010)	<0.001	
>75	223/527	Ref	3.413 (2.059, 6.555)	<0.001	7.321 (4.151, 12.912)	<0.001	
Gender							0.360
Female	124/400	Ref	2.434 (1.299, 4.560)	0.005	4.604 (2.353, 9.006)	<0.001	
Male	238/666	Ref	4.122 (2.481, 6.850)	<0.001	9.639 (5.540, 16.770)	<0.001	
Hypertension							0.117
Yes	202/551	Ref	3.134 (1.913, 5.134)	<0.001	5.938 (3.415, 10.325)	<0.001	
No	160/515	Ref	4.192 (2.134, 8.233)	<0.001	10.308 (5.095, 20.855)	<0.001	
Diabetes							0.897
Yes	101/287	Ref	3.913 (1.899, 8.072)	<0.001	6.265 (2.845, 13.795)	<0.001	
No	261/779	Ref	3.223 (2.014, 5.518)	<0.001	7.607 (4.584, 12.622)	<0.001	
SOFA score							0.508
<=12	133/608	Ref	7.792 (2.850, 21.304)	<0.001	5.500 (2.261, 13.380)	<0.001	
>12	229/458	Ref	4.509 (1.937, 10.501)	<0.001	3.125 (1.377, 7.090)	0.005	
AKI							0.761
Yes	214/483	Ref	4.597 (2.408, 8.776)	<0.001	6.802 (3.501, 13.213)	<0.001	
No	148/583	Ref	2.462 (1.485, 4.084)	<0.001	6.389 (3.521, 11.590)	<0.001	

P1: Middle N/LP group vs Low N/LP group; P2: High N/LP group vs Low N/LP group; SOFA, sequential organ failure assessment; AKI, acute kidney injury.

### ROC analysis of the predictive value of the N/LP ratio for 30/60 days mortality and incidence of AKI

**Figure 8:**
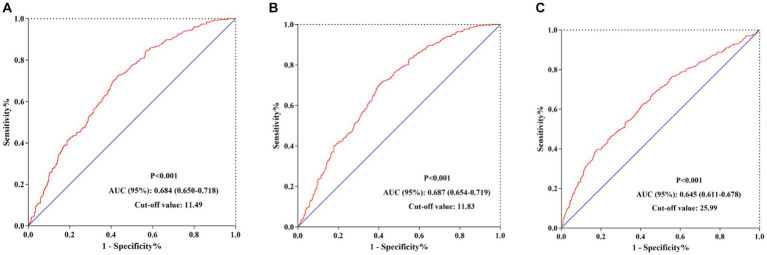
The predictive value of N/LP ratio for 30 days/60 days mortality and AKI occurrence by ROC analysis. **(A)** The predictive value of N/LP ratio for 30 days mortality by ROC analysis; **(B)** The predictive value of N/LP ratio for 60 days mortality by ROC analysis; **(C)** The predictive value of N/LP ratio for AKI occurrence by ROC analysis. ROC, receiver operating characteristic curve; AUC, area under curve; AKI, acute kidney injury.

We conducted AUC analyses to evaluate the predictive value of the N/LP ratio and other relevant indicators. The N/LP ratio exhibited a higher AUC (0.684, 95% CI: 0.650, 0.718) for predicting 30 days mortality, with a cut-off value of 11.49 (Figure 8A). In comparison, the AUC values for NLR, WBC, Neu, Lym, PLT, CRP, Alb, creatinine, glucose, BUN, uric acid, PCT, D-dimer, and lactate level were 0.641, 0.537, 0.548, 0.629, 0.621, 0.548, 0.559, 0.635, 0.530, 0.681, 0.631, 0.511, 0.621, and 0.675, respectively (Table 5). The sensitivity and specificity for predicting 30 days mortality using the N/LP ratio were 72.5 and 56.7%, respectively. Similarly, the N/LP ratio also demonstrated a higher AUC (0.687, 95% CI: 0.654, 0.719) for predicting 60 days mortality compared to the other indicators (Figure 8B). The AUC values for NLR, WBC, Neu, Lym, PLT, CRP, Alb, creatinine, glucose, BUN, uric acid, PCT, D-dimer, and lactate level were 0.652, 0.543, 0.557, 0.633, 0.608, 0.547, 0.569, 0.618, 0.540, 0.666, 0.596, 0.499, 0.617, and 0.676, respectively (Table 5). The ROC analysis identified a cut-off value of 11.83 for the N/LP ratio, with a sensitivity of 70.7% and specificity of 59.4% in predicting 60 days mortality. Furthermore, we found that the N/LP ratio was also predictive of the incidence of AKI, with an AUC value of 0.645 (95% CI: 0.611, 0.678) and a cut-off value of >25.99 (Figure 8C). The sensitivity and specificity for predicting AKI using the N/LP ratio were 39.5 and 82.3%, respectively (Supplementary Table S2).

**Table 5:** The diagnostic accuracy of various prediction factors for 30/60 days mortality.

Variables	30 days mortality					60 days mortality				
	AUC (95%)	Cut-off value	Sensitivity	Specificity	*p*	AUC (95%)	Cut-off value	Sensitivity	Specificity	*p*
N/LP	0.684 (0.650, 0.718)	11.49	0.725	0.567	<0.001	0.687 (0.654, 0.719)	11.83	0.707	0.594	<0.001
NLR	0.641 (0.606, 0.676)	17.82	0.680	0.571	<0.001	0.652 (0.619, 0.686)	16.11	0.718	0.534	<0.001
WBC *10^9^/L	0.537 (0.499, 0.575)	12.55	0.497	0.578	0.056	0.543 (0.507, 0.579)	12.55	0.494	0.582	0.021
Neu *10^9^/L	0.548 (0.511, 0.586)	8.45	0.670	0.416	0.013	0.557 (0.521, 0.593)	7.85	0.704	0.389	0.002
Lym *10^9^/L	0.629 (0.593, 0.664)	0.9	0.856	0.330	<0.001	0.633 (0.599, 0.667)	0.9	0.854	0.344	<0.001
PLT *10^9^/L	0.621 (0.583, 0.658)	152	0.644	0.546	<0.001	0.608 (0.572, 0.644)	146	0.588	0.588	<0.001
CRP, mg/L	0.548 (0.511, 0.585)	30.90	0.882	0.236	0.014	0.547 (0.512, 0.583)	34.40	0.865	0.260	0.011
Alb, g/L	0.559 (0.520, 0.598)	27.15	0.533	0.610	0.002	0.569 (0.533, 0.606)	27.15	0.530	0.620	<0.001
Creatinine, μmol/L	0.635 (0.598, 0.673)	121.20	0.549	0.695	<0.001	0.618 (0.582, 0.655)	121.20	0.530	0.705	<0.001
Glucose, mmol/L	0.530 (0.491, 0.569)	15.78	0.154	0.916	0.121	0.540 (0.503, 0.578)	10.59	0.357	0.720	0.031
BUN, mmol/L	0.681 (0.646, 0.716)	11.68	0.529	0.749	<0.001	0.666 (0.632, 0.700)	11.68	0.503	0.757	<0.001
Uric acid, μmol/L	0.631 (0.593, 0.668)	458.55	0.337	0.880	<0.001	0.596 (0.558, 0.633)	456.55	0.312	0.882	<0.001
PCT, ng/mL	0.511 (0.454, 0.568)	4.48	0.527	0.537	0.697	0.499 (0.444, 0.553)	34.50	0.216	0.836	0.957
D-dimer, mg/L	0.621 (0.583, 0.659)	5.27	0.575	0.641	<0.001	0.617 (0.581, 0.652)	4.23	0.635	0.565	<0.001
Lactate level, mmol/L	0.675 (0.638, 0.712)	2.1	0.703	0.587	<0.001	0.676 (0.641, 0.712)	2.1	0.688	0.603	<0.001

NLR, neutrophil-to-lymphocyte ratio; WBC, white blood cell; Neu, neutrophil; Lym, lymphocyte; PLT, platelet; CRP, C-reactive protein; Alb, albumin; BUN, blood urea nitroge; PCT, procalcitonin; AUC, area under curve.

### Association between N/LP ratios and other secondary outcomes

Univariate analysis revealed that the high N/LP group was significantly associated with an increased risk of CRRT (*p* < 0.001), vasoactive drug usage (*p* < 0.001), longer ICU stay (*p* < 0.001), and higher ICU mortality rate (*p* < 0.001) when compared to the other groups (Table 2). After adjusting for confounding factors, such as age, sex, hypertension, diabetes, WBC, CRP, Alb, creatinine, BUN, uric acid, PCT, D-dimer, and lactate level, both the middle and high N/LP groups showed a greater risk of ICU mortality with a 2.451/3.830-fold increase (95%CI: 1.273, 4.718; 95%CI: 1.934, 7.585, respectively) compared to the low NLR group. In addition, only the high N/LP group exhibited a significantly increased risk of vasoactive drug usage (95%CI: 1.090, 4.435) and longer ICU stay (95%CI: 0.921, 3.457). However, the N/LP ratio was not found to be predictive of CRRT, invasive ventilation, or length of hospital stay (Table 6).

**Table 6:** Relationship between different N/LP ratios and secondary outcomes.

Variables	Low N/LP group (N/LP < =5.68)	Middle N/LP group (5.68 < N/LP < 28.41)	P1	High N/LP group (N/LP > =28.41)	P2
CRRT, *n* (%)	Ref	2.108 (0.524, 8.478)	0.294	3.049 (0.730, 12.740)	0.127
Vasoactive drug, *n* (%)	Ref	0.982 (0.579, 1.667)	0.946	2.198 (1.090, 4.435)	0.028
Invasive ventilation, *n* (%)	Ref	1.074 (0.634, 1.819)	0.791	0.995 (0.535, 1.852)	0.988
Length of ICU stay, days	Ref	1.287 (−0.658, 3.231)	0.194	2.189 (0.921, 3.457)	0.001
Length of hospital stay, days	Ref	−0.804 (−4.267, 2.658)	0.648	0.536 (−1.774, 2.847)	0.648
ICU mortality, *n* (%)	Ref	2.451 (1.273, 4.718)	0.007	3.830 (1.934, 7.585)	<0.001

P1: Middle N/LP group vs Low N/LP group; P2: High N/LP group vs Low N/LP group; CRRT, continuous renal replacement therapy; ICU, intensive care unit.

## Discussion

In the present study, we had employed various methods to evaluate the association between N/LP ratios and adverse outcomes in sepsis patients. Our findings indicated that the initial N/LP ratio was an independent predictor of 30/60 days mortality and the incidence of AKI after ICU admission. Moreover, we observed that these impacts were more pronounced among male patients, older individuals, and those with a poor health status. Furthermore, we found that the initial N/LP ratio at ICU admission outperforms other biomarkers, such as NLP, WBC, Neu, Lym, PLT, CRP, Alb, creatinine, glucose, BUN, uric acid, PCT, D-dimer, and lactate, in predicting 30/60 days mortality. Additionally, we observed that N/LP measured at sepsis prognosis also exhibited good predictive power for AKI occurrence. Finally, a higher N/LP ratio was found to be strongly associated with disease severity in sepsis patients, including the need for vasoactive drugs, length of ICU stay, and ICU mortality.

Sepsis, a leading cause of serious complications and death in critically ill patients, poses challenges in terms of prevention (24). Early recognition of sepsis is crucial for timely intervention and improving prognosis. Recent evidence suggested that inflammatory response, neutrophil extracellular traps, inflammatory bodies, apoptosis, and endothelial cell dysfunction may be the underlying mechanisms responsible for sepsis development (12, 25–28). Sepsis triggers a systemic cytokine-chemokine response, leading to extensive activation and dysfunction of the immune system. This immune system dysregulation is often manifested as an increase in neutrophilia and decrease in lymphopenia (29). The NLR, calculated from whole blood counts, serves as a biomarker that reflects the interaction between the innate immune system (neutrophils) and adaptive immune system (lymphocytes) (20). Previous studies had highlighted the significance of the NLR in predicting outcomes for a range of inflammation-associated and physiological stress-induced diseases, such as COPD, cancer, and kidney diseases (30–32). Specifically, extensive research had been conducted on the correlation between NLR and adverse outcomes in community-acquired pneumonia (CAP) (33, 34). In the context of CAP, NLR had emerged as a powerful tool for predicting prolonged hospitalization, ICU admission, and even mortality. Notably, a study by Regolo et al. had investigated the association between NLR and adverse outcomes in COVID-19 patients (35, 36). Their findings underscored the importance of NLR as a significant predictor of disease progression and mortality in individuals affected by COVID-19. While there was evidence suggesting an association between NLR and mortality in sepsis patients, the relationship between NLR and clinical prognosis remained a topic of debate (37). In a study by Schupp et al., NLR was found to be a reliable diagnostic tool for identifying patients with sepsis, but no association with an increased risk of 30 days all-cause mortality was observed (38). Similarly, recent research had indicated that there was no relationship between NLR and patient outcomes in sepsis at ICU admission (39). These findings contradicted previous studies conducted on oncology patients with sepsis, where NLR showed promise as a prognostic marker (40).

One possible explanation for these conflicting results is the intricate interplay between immune dysregulation, inflammatory response, and coagulation dysfunction, leading to microvascular dysfunction, platelet activation, and microthrombosis, ultimately resulting in endothelial cell dysfunction (41, 42). To increase the sensitivity of predicting patient outcomes, platelet count has been incorporated into the ratio, given its close association with the inflammatory response and coagulation. Subsequently, Koo et al. developed a combined ratio of N/LP to predict its association with mortality after cardiovascular surgery (16). Additionally, numerous studies had validated the efficiency of a rising N/LP ratio as a predictor of in-hospital mortality in COVID-19 patients and those undergoing emergency surgery (14, 43). However, limited research had been conducted on the association between N/LP ratios and mortality in sepsis patients. In the present study, we found that an elevated N/LP ratio in sepsis patients was independently correlated with 30/60 days mortality. Moreover, the N/LP ratio at ICU admission outperformed traditional disease markers, such as NLP, WBC, Neu, Lym, PLT, CRP, Alb, creatinine, glucose, BUN, uric acid, PCT, D-dimer, and lactate level, in predicting mortality at 30 and 60 days. These findings suggested that early elevation of the N/LP ratio may serve as a potential predictor of short-term mortality in sepsis patients.

Sepsis is a condition characterized by a systemic inflammatory response to an infectious injury, which often leads to multiple organ dysfunction (44). AKI is a common and serious complication in sepsis patients, with an incidence as high as 50% and a mortality rate of 60 to 70% (45). However, the available treatment options for septic AKI are limited. Previous studies had suggested that traditional biomarkers such as serum creatinine concentrations and urine output were not ideal or reliable indicators for diagnosing septic AKI (46, 47). As a result, researchers had explored several novel biomarkers for early detection of septic AKI, including kidney injury molecule-1 (KIM-1), neutrophil gelatinase-related lipocalin (NGAL), liver-type fatty acid binding protein (L-FABP), tissue inhibitor of metalloproteinase 2 (TIMP2), and insulin-like growth factor binding protein 7 (IGFBP7) (48–52). However, most of these biomarkers still require external validation and further clinical investigation. In recent years, the N/LP ratio had emerged as an economically viable and widely available biomarker associated with kidney function (53). A recent retrospective study demonstrated a significant association between varying N/LP ratios and the occurrence of AKI following abdominal and cardiovascular surgery (15, 16). In our current study, we observed that elevated N/LP ratios in sepsis patients were associated with an increased risk of AKI occurrence, which was consistent with a previous report on AKI patients in the ICU (13). Furthermore, our study revealed that the initial N/LP ratio measured at ICU admission can serve as a predictive factor for the occurrence of AKI. These findings indicated that evaluating the N/LP ratio could potentially enable earlier diagnosis of AKI, which in turn may contribute to a reduction in AKI incidence.

In this study, we aimed to investigate the association between N/LP ratios and adverse outcomes. The severity of illness and risk of mortality in critically ill patients are commonly assessed using the SOFA score and APACHE II score (54, 55). Previous studies had consistently reported that high SOFA or APACHE II scores were independent predictors of poor prognosis in sepsis patients (56, 57). We performed correlation analyses to examine the relationship between N/LP ratios and SOFA score and APACHE II score, and our findings revealed a statistically significant positive correlation. In relation to in-hospital events, an elevated N/LP ratio at the time of ICU admission emerged as a valuable inflammatory marker for evaluating the need for vasoactive drugs, prolonged ICU stays, and the risk of ICU mortality in sepsis patients. These findings underscored the significance of the initial N/LP value upon ICU admission in facilitating the early identification of adverse outcomes. Consequently, it is crucial to provide timely intervention for patients with a high N/LP value to prevent further deterioration.

### Strengths and limitations

Our study had several strengths. The study was one of the first to investigate the association between N/LP ratios and adverse outcomes in sepsis patients, contributing new knowledge to the field. Various regression models were used to analyze the effect of N/LP ratio on sepsis patients, increasing the robustness of the findings. The study utilized variables that were part of daily clinical practice, enhancing the generalizability of the results. The results were reliable and provide a foundation for clinical diagnosis and intervention in sepsis patients. However, this study had several limitations. First, the study was limited by its retrospective nature, which introduced the possibility of selection bias and affects the causal interpretation of the findings. Second, only the impact of the N/LP ratio on 30/60 days mortality and occurrence of AKI in sepsis patients was analyzed, limiting the scope of the study’s findings. Third, the lack of recorded prehospital interventions that could affect the N/LP ratio reduces the comprehensiveness of the analysis. Moreover, the study only examined the N/LP ratio within 24 h of ICU admission, failing to capture the dynamic changes in N/LP ratio over time in sepsis patients. Additionally, data of this study cannot be extrapolated all over the world because of race differences. Finally, due to the lack of some data regarding blood gas analyses, data on PaO_2_/FiO_2_ (P/F) were not shown, so deppriving the statistical analysis of an important variable extremely useful to assess the severity of respiratory failure. To further validate and strengthen the findings, it is recommended to design a large multicenter prospective study that addresses the limitations mentioned above. This would provide more robust evidence regarding the association between N/LP ratio and sepsis outcomes.

## Conclusion

This retrospective study provided evidence that an elevated N/LP ratio at ICU admission was associated with a higher risk of 30/60 days mortality and the incidence of AKI in sepsis patients. The N/LP ratio showed potential as a useful biomarker for predicting these outcomes. Furthermore, the impact of N/LP ratio on outcomes appeared to be stronger in male patients, older individuals, and those with poorer health status. However, it is important to note that further research is needed to validate these findings, preferably through large-scale multicenter prospective studies.

## Data availability statement

The raw data supporting the conclusions of this article will be made available by the authors, without undue reservation.

## Ethics statement

The studies involving humans were approved by the Affiliated Hospital of Jiangsu University (Approval No. KY2023K1007). The studies were conducted in accordance with the local legislation and institutional requirements. The participants provided their written informed consent to participate in this study. Written informed consent was obtained from the individual(s) for the publication of any potentially identifiable images or data included in this article.

## Author contributions

JZ: Conceptualization, Data curation, Formal analysis, Writing – original draft, Writing – review & editing. QZ: Conceptualization, Formal analysis, Writing – review & editing. ZH: Conceptualization, Formal analysis, Supervision, Writing – original draft, Writing – review & editing.
