# Supplementary material for: Clinical predictive value of the initial neutrophils to lymphocytes and platelets ratio for prognosis of sepsis patients in the intensive care unit: a retrospective study
Source: Front Med (Lausanne). 2024 Jan 18;11:1351492. doi: 10.3389/fmed.2024.1351492 (PMC10840849; doi:10.3389/fmed.2024.1351492)
Supplement: Supplementary file 1 [file Table_1.docx]

**Supplemental Materials**

**Supplemental Table 1.** Subgroup analysis regarding the influence of different N/LP ratios in the AKI occurrence.

**Supplemental Table 2.** The diagnostic accuracy of various prediction factors for the AKI occurrence.

**Supplemental Table 1**. Subgroup analysis regarding the influence of different N/LP ratios in the AKI occurrence.

| **Subgroups** | **No.AKI/No.patients** | **Low N/LP group**  **(N/LP<=5.68)** | **Middle N/LP group**  **(5.68<N/LP<28.41)** | **P1** | **High N/LP group**  **(N/LP>=28.41)** | **P2** | **P for interaction** |
| --- | --- | --- | --- | --- | --- | --- | --- |
| **Age** |  |  |  |  |  |  | 0.982 |
| <=75 | 229/539 | Ref | 1.471 (0.946, 2.285) | 0.086 | 3.980 (2.401, 6.600) | <0.001 |  |
| >75 | 254/527 | Ref | 1.823 (1.175, 2.831) | 0.007 | 4.096 (2.443, 6.687) | <0.001 |  |
| **Gender** |  |  |  |  |  |  | 0.110 |
| Female | 187/400 | Ref | 1.293 (0.776, 2.154) | 0.324 | 5.868 (3.192, 10.787) | <0.001 |  |
| Male | 296/666 | Ref | 1.889 (1.275, 2.796) | 0.001 | 3.246 (2.062, 5.111) | <0.001 |  |
| **Hypertension** |  |  |  |  |  |  | 0.237 |
| Yes | 263/551 | Ref | 1.587 (1.046, 2.409) | 0.029 | 5.369 (3.198, 9.011) | <0.001 |  |
| No | 220/515 | Ref | 1.739 (1.088, 2.780) | 0.020 | 3.308 (1.963, 5.575) | <0.001 |  |
| **Diabetes** |  |  |  |  |  |  | 0.799 |
| Yes | 151/287 | Ref | 1.739 (0.986, 3.068) | 0.055 | 4.624 (2.301, 9.292) | <0.001 |  |
| No | 332/779 | Ref | 1.647 (1.133, 2.396) | 0.009 | 3.970 (2.588, 6.090) | <0.001 |  |
| **SOFA score** |  |  |  |  |  |  | 0.208 |
| <=12 | 216/608 | Ref | 1.698 (0.869, 3.317) | 0.120 | 1.186 (0.723, 1.946) | 0.499 |  |
| >12 | 267/458 | Ref | 3.196 (1.476, 6.920) | 0.003 | 1.909 (0.935, 3.900) | 0.074 |  |
| **Lactate level** |  |  |  |  |  |  | 0.415 |
| <=2.0 | 162/514 | Ref | 1.326 (0.846, 2.076) | 0.217 | 4.170 (2.363, 7.356) | <0.001 |  |
| >2.0 | 321/552 | Ref | 1.725 (1.092, 2.727) | 0.019 | 2.848 (1.732, 4.683) | <0.001 |  |

P1: Middle N/LP group vs Low N/LP group; P2: High N/LP group vs Low N/LP group;

Abbreviations: SOFA, Sequential Organ Failure Assessment; AKI, Acute kidney injury.

**Supplemental Table 2**. The diagnostic accuracy of various prediction factors for the AKI occurrence.

| Variables |  | AKI |  | | |
| --- | --- | --- | --- | --- | --- |
|  | AUC (95%) | Cut-off value | sensitivity | specificity | P |
| N/LP | 0.645 (0.611, 0.678) | 25.99 | 0.395 | 0.823 | <0.001 |
| NLR | 0.603 (0.569, 0.637) | 20.29 | 0.553 | 0.643 | <0.001 |
| WBC *10^9^ /L | 0.575 (0.540, 0.610) | 12.55 | 0.522 | 0.621 | <0.001 |
| Neu *10^9^ /L | 0.582 (0.547, 0.617) | 11.15 | 0.538 | 0.617 | <0.001 |
| Lym *10^9^ /L | 0.561 (0.527, 0.596) | 0.7 | 0.669 | 0.463 | 0.001 |
| PLT *10^9^ /L | 0.627 (0.593, 0.660) | 123 | 0.476 | 0.727 | <0.001 |
| CRP, mg/L | 0.625 (0.591, 0.659) | 121.50 | 0.528 | 0.695 | <0.001 |
| Alb, g/L | 0.565 (0.530, 0.600) | 27.75 | 0.547 | 0.596 | <0.001 |
| Glucose, mmol/L | 0.575 (0.540, 0.610) | 12.15 | 0.301 | 0.842 | <0.001 |
| D-dimer, mg/L | 0.662 (0.629, 0.695) | 5.24 | 0.566 | 0.694 | <0.001 |
| Lactate level, mmol/L | 0.694 (0.661, 0.727) | 2.8 | 0.510 | 0.774 | <0.001 |

Abbreviations: NLR, neutrophil-to-lymphocyte ratio; WBC, white blood cell; Neu, neutrophil; Lym, lymphocyte; PLT, platelet; CRP, C-reactive protein; Alb, albumin; AUC, Area Under Curve; AKI, Acute kidney injury.
